# Medication Monitoring in a Nurse-Led Respiratory Outpatient Clinic: Pragmatic Randomised Trial of the West Wales Adverse Drug Reaction Profile

**DOI:** 10.1371/journal.pone.0096682

**Published:** 2014-05-05

**Authors:** Marie E. Gabe, Fiona Murphy, Gwyneth A. Davies, Ian T. Russell, Susan Jordan

**Affiliations:** 1 College of Human and Health Sciences, Swansea University, Swansea, United Kingdom; 2 ABM University Health Board, Wales, United Kingdom; 3 College of Medicine, Swansea University, Swansea, United Kingdom; 4 West Wales Organisation for Rigorous Trials in Health, and College of Medicine, Swansea University, Swansea, United Kingdom; Baylor College of Medicine, United States of America

## Abstract

**Objective:**

To assess the clinical effect of medication monitoring using the West Wales Adverse Drug Reaction (ADR) Profile for Respiratory Medicine.

**Design:**

Single-site parallel-arm pragmatic trial using stratified randomisation.

**Setting:**

Nurse-led respiratory outpatient clinic in general hospital in South Wales.

**Participants:**

54 patients with chronic respiratory disease receiving bronchodilators, corticosteroids or leukotriene receptor antagonists.

**Intervention:**

Following initial observation of usual nursing care, we allocated participants at random to receive at follow up: either the West Wales ADR Profile for Respiratory Medicine in addition to usual care (‘intervention arm’ with 26 participants); or usual care alone (‘control arm’ with 28 participants).

**Main Outcome Measures:**

Problems reported and actions taken.

**Results:**

We followed up all randomised participants, and analysed data in accordance with treatment allocated. The increase in numbers of problems per participant identified at follow up was significantly higher in the intervention arm, where the median increase was 20.5 [inter-quartile range (IQR) 13–26], while that in the control arm was −1 [−3 to +2] [Mann-Whitney U test: z = 6.28, p<0.001]. The increase in numbers of actions per participant taken at follow up was also significantly higher in the intervention arm, where the median increase was 2.5 [1]–[4] while that in the control arm was 0 [−1.75 to +1] [Mann-Whitney U test: z = 4.40, p<0.001].

**Conclusion:**

When added to usual nursing care, the West Wales ADR Profile identified more problems and prompted more nursing actions. Our ADR Profile warrants further investigation as a strategy to optimise medication management.

**Trial Registration:**

Controlled-trials.com ISRCTN10386209

## Introduction

Iatrogenic harm is a persistent concern [1]. No pharmacological therapy that is effective is free of adverse effects [2]. Adverse drug reactions (ADRs), defined as any untoward and unintended response in a patient or investigational subject to a medicinal product that relates to any dose administered [3], range from minor and reversible through disability to death. Preventable ADRs account for 3.7% of hospital admissions in developed countries [4], and generate a 4.7% mortality rate in hospital [5]. Five per cent of inpatients experience ADRs [6], rising to 14% in elderly care wards [7]. Thus, this issue causes international concern. In the UK, about 6.5% of hospital admissions are due to ADRs, most of which are preventable [8], [9]; however we know less about the prevalence of ADRs in the community not associated with hospital admission [10].

ADRs are an important source of patient harm and healthcare expenditure. However there is significant heterogeneity between studies in risk of bias, populations, settings and methods used to identify ADRs [8], [11]–[13]. In the US, ADRs are recognised in the top six causes of death [14], costing $370 per ADR case for emergency department visits [15]. The cost of each ADR case is estimated at €2250 in Germany [16]. In the UK, ADR-related admissions alone cost the NHS an estimated £466 m *per annum* [10]; however, this estimate does not account for non-hospital contact [17]. Variability in identifying, recording and reporting of adverse events and ADRs accentuates this public health problem. Improved monitoring of, and response to, identified ADRs is an international priority to reduce the unnecessary burden of treatment, yet remains under-researched [18]–[22].

### Background

There are underlying weaknesses in current medication management practice internationally, particularly medication monitoring for known ADRs of prescribed drugs [18], [20], [21], [23]–[25]. Healthcare systems have traditionally relied on patient and clinician self-reports, prescription-event monitoring and case-control studies. These approaches capture serious and unexpected ADRs, whilst minor to moderate ADRs are typically less well reported [26] but remain burdensome to service users. Despite the advantages of service user ADR reporting [27], under-reporting is widely recognised: in a clinical trial only 1.5–5.1% (5/328-11/216) of ADR-related problems were spontaneously reported to physicians, increasing to 29–49% (96/328-105/216) when a questionnaire on specific known ADRs was administered [28], and the overall ADR reporting rate may be around 1% [29]. A survey of 661 outpatients in Boston identified that 37% of ameliorable ADRs were related to failure of service users to inform physicians (19/51) [30]. Public awareness of ADR reporting remains low [31], [32]: a survey of 2028 UK adults indicated that while 477 (24%) had experienced an ADR, only 172/2028 (9%) were aware of reporting mechanisms [33], [34]. ADR reporting by physicians may retain an element of subjectivity [35], [36], and the long-term effects of educational interventions to increase ADR reporting are unclear [37]. Further investment is needed to develop feasible evidence-based strategies, that engage service users [32], and are not overly time consuming, and explore their clinical impact [18], [19], [20], [23], [24], [38]–[43].

### Respiratory Medicine

Thirty five million Americans live with chronic lung disease [44], and the financial burden of lung disease in Europe is estimated at €100 billion, of which, €6.7 billion is prescription drug costs [45]. In the UK, almost 8 million individuals suffer with lung disease, accounting for 7% of all drugs prescribed [46]. Inhaled beta-agonists, often prescribed in tandem with corticosteroids, are among the top ten drugs causing ADRs [47]. However, no comprehensive and validated medication monitoring profile in respiratory medicine has been located. Guidelines on the management of chronic respiratory diseases are available [48], [49], but do not describe medication monitoring.

To address this deficit, the West Wales ADR (WWADR) Profile for respiratory medicine has been developed from existing literature summarising adverse effects [50]–[53], and tested for cognitive form, validity and reliability [22], [54]. This paper sets the foundation for larger trials of nurse-led medication monitoring initiatives.

## Methods

The protocol for this trial and supporting CONSORT checklist are available as supporting information, see Checklist S1 and Protocol S1.

### Ethics Statement

Favourable ethical opinion for this study was received from the South West Wales Research Ethics Committee (February 2010 reference 09/WMW02/60). This allowed the study to take place in the National Health Service (NHS) in South West Wales. The University, as trial sponsors, accepted this decision, and did not seek further review. Patient information sheets were available in English and Welsh ahead of gaining informed written consent from all participants. All participant data were anonymised.

### Aim

We aimed to explore the clinical effect of nurse-led medication monitoring using the West Wales ADR (WWADR) Profile for Respiratory Medicines. Key outcomes were changes in problems found and actions taken during nurse-patient consultations before and after introduction of the ADR Profile.

### Setting

A nurse-led outpatient respiratory clinic in a large 600-bed general hospital in South West Wales, that serves an urban and rural population of around 600,000.

### Design

We undertook a single-site parallel-arm pragmatic randomised trial with stratified random allocation performed by West Wales Organisation for Rigorous Trials in Health (WWORTH), the local clinical trials unit [table 1]. At observation 1, all patients received usual outpatient care, which included checking patients’ medication, symptoms, health and wellbeing. At their next scheduled outpatient visit, observation 2, the control arm received usual care only, while the intervention arm, in addition to usual care, received medication monitoring with the ADR Profile. The researcher observed all consultations. The interval between the first and second round of observations was dictated by the nurses’ clinical judgment, based on participants’ needs.

**Table 1:** Trial design.

	Clinic visit 1 and data collection 1	Interval between clinic visits	Clinic visit 2 and datacollection 2
**Intervention arm**	Usual care +Observation of 26 consultations	Usual care	Usual care **+ WWADR** **Profile** + Observationof 26 consultations
**Control arm**	Usual care + Observationof 28 consultations	Usual care	Usual care +Observation of 28 consultations

### The Intervention

The WWADR Profile for Respiratory Medicine was used in the intervention arm at observation two [table 1] by the respiratory nurse specialists working in the outpatient clinic. The WWADR Profile [54] is a five-sectioned ADR symptom questionnaire uniting problem detection with actions over five sections comprising 81 items (57 questions, 4 observations, 7 measurements, 13 checks of laboratory data), covering symptoms of the known ADRs of corticosteroids, bronchodilators and leukotriene receptor antagonists. We derived these items from the literature on ADRs [22], [50]–[53], invited service users and expert clinicians to scrutinise them for relevance, feasibility and accuracy, and investigated validity and reliability [54]. Targeted questions seek to identify problems that may be indicative of ADRs, and merit attention regardless of aetiology. For example: muscular weakness may be symptomatic of steroid-induced metabolic changes or hypokalaemia [50]–[52], [55]; reports of mood changes may indicate steroid-induced mood disorders [56]–[58] or hypokalaemia [51]–[53]. The Profile also includes health promotion advice that is particularly pertinent to users of respiratory medicines. For example, high salt intake may exacerbate steroid-induced fluid retention [52]. The risk of adverse reactions is reduced, but not eliminated, when inhaled preparations are used [53].

### Randomisation

We undertook remote randomisation after the first round of observations. We had selected five stratification variables from the literature and previous work [54]: age (more or less than median of 61 years), number of respiratory medicines (more or less than median of two), number of other drugs prescribed (more or less than median of five), sex and day of initial observation (Wednesday or Friday clinic). We passed participant study numbers and stratification variables to WWORTH by telephone. They used a dynamic randomisation algorithm based on weighted squared-difference measures of imbalance to allocate participants in the ratio of 1∶1 and balance the five stratification variables without blocking [59]. We used hospital numbers to track participants’ return to clinic for the second round of observations.

### Participants

Patients were screened by their consultant and nurses to ensure they were well enough to participate. All eligible patients were approached in respiratory outpatient clinics January 2011–January 2012.

Inclusion Criteria:

- Established clients of the nurse-led outpatient respiratory clinic
- Existing chronic respiratory condition
- Currently prescribed at least one of: bronchodilators, corticosteroids or leukotriene receptor antagonists

Exclusion Criteria:

- Aged 16 or under
- Lack of fluency in English or Welsh
- Considered by their nurses or consultant to lack the capacity to consent, or to be unduly stressed or vulnerable (for example the recently bereaved).

### Outcome Measures

Primary outcomes:

1. Change in numbers of problems found per participant between observations one and two
2. Change in numbers of actions taken per participant between observations one and two

Secondary outcomes:

1. Numbers with at least one action taken at observation two
2. Numbers of actions taken per participant at observation two

### Data Collection and Measurement of Outcomes

Data on problems identified and actions taken were collected for each participant at both observations using a structured template of possible ADRs [54] [table 1]. The nurse completed an independent copy of the WWADR Profile for the intervention participants. In addition, the researcher kept freehand field notes, primarily to indicate all nursing actions taken. Nurses and patients were asked to comment on use of the Profile and identify possible harm to patients.

For outcome 2.1, ADR actions were distinguished from all actions: ADR actions were those directly prompted by the WWADR Profile whilst ‘all actions’ also included those identified from usual care, or discussion around questions in the WWADR Profile.

### Sample Size

Previously, 14/20 patients receiving usual care and 1/20 patients responding to a structured monitoring instrument had no ADRs on a monitoring profile addressed by their nurses [60], [61]. A sample of 34, 17 in each arm, is sufficient to detect this difference in incidence of nursing actions with 90% power when using 1% significance level [62]. To allow for potential losses to follow up within available resources, the sample was inflated to 54.

### Blinding

At observation one, the nurse, researcher and participants were unaware of intended allocation. However, at observation two, participants, based on their previous experience in clinic, might have guessed that they were receiving the intervention. Nurses were unblinded when delivering the intervention. The researchers were aware of allocation during observation, but were blinded for analysis.

### Data Analysis

Data were analysed using IBM SPSS statistics version 19. Data were double entered and checked for data entry errors. No mistakes were identified. To test whether data were normally distributed we used the Shapiro-Wilk test for samples smaller than 50 and the Kolmogorov-Smirnov test for samples larger than 50. To compare between arms those variables found not to be normally distributed we used the Mann-Whitney U test, the non-parametric equivalents of the t test. Categorical variables were tested by chi squared tests or Fisher’s exact test and logistic regression using the backward likelihood ratio criterion. Using ‘increased number of ADR-related actions taken at observation two’ as the outcome variable, arm allocation, age, sex and consultation two duration were entered into univariate models as sole predictor variables. Arm allocation was then tested with each of the four other variables iteratively. Participants’ comments are included for illustration.

## Results

**Figure 1:**
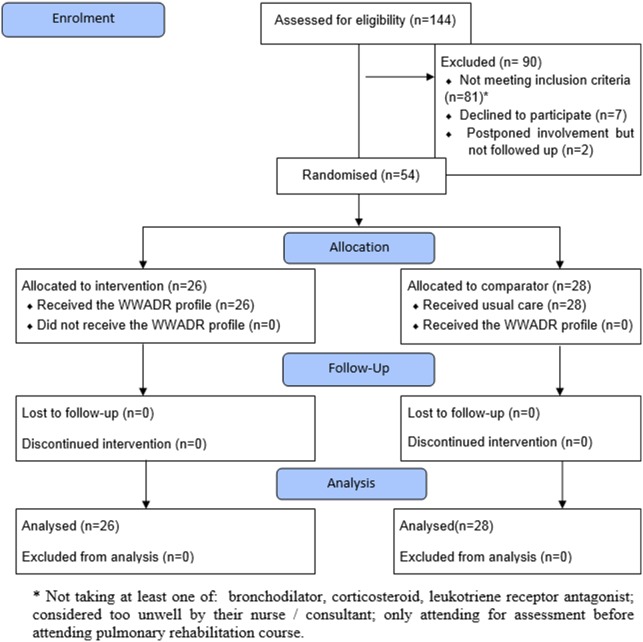
Flow of participants through the trial.

A total of 63 eligible patients were seen in clinic, 54 were recruited (86%). No losses occurred and all participants were analysed in accordance with their original allocation [figure 1]. Control participants were asked to attend on Wednesdays and intervention participants on Fridays. Although 18 participants attended on the ‘incorrect’ day, all received the treatment allocated.

Some interval variables were not normally distributed, necessitating non-parametric testing. Table 2 shows that the trial arms, comparable at the first consultation, differed in the length of the second consultation.

**Table 2:** Participant and consultation characteristics.

	Control arm (n = 28, unless otherwise stated)	Intervention arm (n = 26)
Number male (%)	13 (46%)	13 (50%)
Mean age at observation one in years (SD)	63.71 (13.6)	62.38 (10.3)
Mean number of respiratory drugs* at observation one (SD)	2.29 (0.8)	2.12 (0.6)
Mean number of respiratory drugs* at observation two (SD)	2.36 (0.8)	2.12 (0.6)
Mean consultation one length, minutes (SD)	14.75 (9.8)	13.85 (8.6)
Mean consultation two length, minutes (SD)	9.15 (9.8) (n = 27)	19.19 (8.7)
Interval between observations 1 and 2 in weeks (SD)	18.39 (10.1)	17.85 (10.8)

*Those in the inclusion criteria plus theophylline.

### Outcomes 1.1 & 1.2. Changes in Numbers of Problems Found and Actions taken per Participant between Observations One and Two

The change in numbers of problems found per participant between observations one and two was significantly different (intervention arm median 20.5, [inter-quartile range (IQR) 13 to 26]; control arm median −1, [IQR −3 to 2]: Mann-Whitney U 1.50, z 6.28, p<0.001) [table 3]. The change in all actions taken was also significant (intervention arm median 2.5, [IQR 1 to 4]; control arm median 0 [−1.75 to +1]: Mann-Whitney U 112.50, z 4.40, p<0.001) [table 3].

**Table 3:** Changes in numbers of problems found and actions taken between observations 1 and 2: Outcomes 1.1 & 1.2.

	Intervention arm		Control arm						
	Median (Inter-quartile range)	Range (min, max)	Median (Inter-quartile range)	Range (min, max)	Mann-Whitney U	Z	Asymp Sig. (2-sided)		r
**Outcome 1.1 Problems found per participant**									
Number of all problems at observation 1	4 (2–6)	9 (1, 10)	4 (3.25–6)	18 (0, 18)					
Number of all problems at observation 2	25 (16–29.75)	30 (7, 37)	4 (2–7)	10 (1, 11)					
Change in the number of all problems/participant	20.5 (13–26)	26 (6, 32)	−1 (−3 - +2)	17 (−10, 7)	1.50	6.28	<0.001	0.85	
**Outcome 1.2 Actions taken per participant**									
Number of all actions at observation 1	2 (0–3)	4 (0, 4)	2 (0.25–2.75)	7 (0, 7)					
Number of all actions at observation 2	4 (3–6)	8 (0, 8)	2 (1–3)	4 (0, 4)					
Change in the number of all actions taken/participant	2.5 (1–4)	8 (−1, 7)	0 (−1.75 - +1)	6 (−3, 3)	112.50	4.40	<0.001	0.60	

### Outcome 2.1. Number of Participants with at Least One Action taken at Observation Two

No significant difference was observed in the numbers of participants with at least one action taken at observations one and two [table 4]. However, the difference in ADR specific actions was significant. At observation two, 1 of 26 (4%) intervention participants had no ADR actions taken, compared with 9 of 28 (32%) controls: *X* ^2^ 5.40, df 1, p 0.02, OR 11.84 (1.38–101.70) [table 4].

**Table 4:** Proportions of participants with at least one action and ADR addressed: Outcome 2.1.

	Interventionarm	Controlarm	*x* ^2^ (df = 1)	Asymp Sig.(2-sided)	Odds ratio(95%CI)	Fisher’sExact testSig. (2-sided)	
**Outcome 2.1. Proportions of participants having at least one action taken in observation one**							
Any actiontaken	19/26 (73.1%)	21/28 (75%)	<0.001	1.00	0.90 (0.26 to 3.06)		
No actiontaken	7/26 (26.9%)	7/28 (25%)					
**Outcome 2.1. Proportions of participants having at least one action taken in observation two**							
Any actiontaken	25/26 (96.2%)	24/28 (85.7%)				0.35	
No actiontaken	1/26 (3.9%)	4/28 (14.3%)					
**Outcome 2.1. Proportions of patients having at least one ADR addressed in observation one**							
Any ADRaction taken	15/26 (57.7%)	19/28 (67.9%)	0.24	0.62	0.65 (0.21 to 1.96)		
No ADRaction taken	11/26 (42.3%)	9/28 (32.1%)					
**Outcome 2.1. Proportions of patients having at least one ADR addressed in observation two**							
Any ADRaction taken	25/26 (96.2%)	19/28 (67.9%)	5.40	0.02	11.84 (1.38 to 101.70)		
No ADRaction taken	1/26 (3.9%)	9/28 (32.1%)					

### Outcome 2.2. Number of Actions taken per Participant at Observation Two

**Figure 2:**
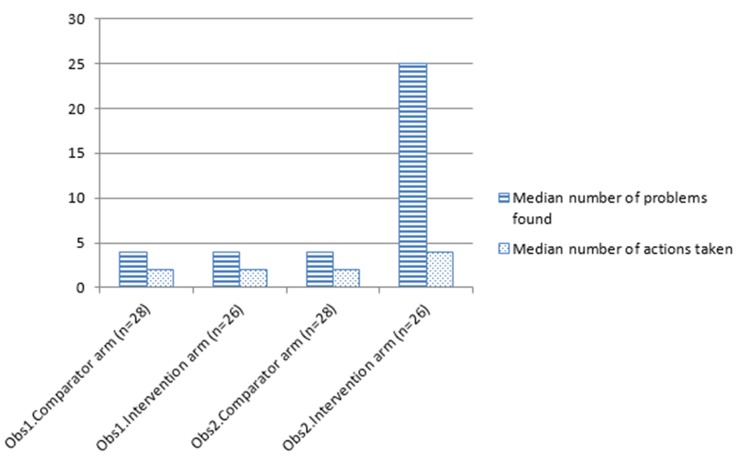
Bar chart of problems found and actions taken par participant at observations one and two.

At observation two, significantly more actions were taken in the intervention arm (median 4, inter-quartile range 3–6) than the control arm (median 2, inter-quartile range 1–3): Mann-Whitney U 112.00, z 4.42, p<0.001, r 0.60 [table 5; figure 2].

**Table 5:** Numbers of actions taken per participant: Outcome 2.2.

	Intervention arm Median (IQR)	Control arm Median (IQR)	Mann-Whitney U	Z	Asymp Sig. (2-sided)	r
**Outcome 2.2. Numbers of actions taken per participant. Observation one**						
All actions	2 (0–3)	2 (0.25–2.75)	362.50	0.03	0.98	0.004
ADR related actions	1 (0–2.25)	2 (0–2)	324.00	0.72	0.47	0.10
**Outcome 2.2. Numbers of actions taken per participant. Observation two**						
All actions	4 (3–6)	2 (1–3)	112.00	4.42	<0.001	0.60
ADR related actions	4 (2–5)	1 (0–2)	84.50	4.91	<0.001	0.67

### Multivariate Analysis

The only significant predictor of increased number of ADR-related actions was arm allocation.

Other predictors were not significant when tested alone or with ‘trial arm’ [Table S1]. Only one participant had a negative result for ‘at least one action taken at observation 2’ and ‘at least one ADR action taken at observation 2’, precluding multivariate analysis.

### Clinical Impact

Problems identified in response to using the WWADR Profile, along with lifestyle factors, included:

- Muscular weakness affecting activities of daily living (22/26, 84.6%), muscular pain (18/26, 69.2%).
- Dry mouth, hoarseness, problems with teeth or dentures, changes in taste or smell of the participants’ breath (19/26, 73.1%).
- Tendency to bruising (17/26, 65.4%), delayed skin healing (16/26, 61.5%). For example: participant 004, who regularly took aspirin and a combined corticosteroid/bronchodilator inhaler, reported “bruises with the slightest knock, sometimes very bad” and attributed this to “just with getting older, normal”.
- Salt regularly added to meals or during cooking (17/26, 65.4%).
- Mood problems (16/26, 61.5%). For example, the nurse commented: “I would not have picked up on participant 014’s depression [without the WWADR Profile]”.
- Fine tremor affecting activities of daily living (12/26, 46.2%). For example: participant 023, who took a high dose of beta agonists, was identified by the ADR Profile as having a fine motor tremor, and commented “they [hands] shake now but I put it down to age… …get annoyed with myself dropping things”.

Actions taken by the participants’ nurses in response to using the WWADR Profile included:

- 32 referrals to other healthcare professionals. Most referrals were to the patients’ primary care practitioners [27/32, 84.4%] for: urinary dysfunction (4); falls/balance problems (2); mood problems (1); prescription of additional medication, for example to treat oral candidiasis (2), suspected infection (3).
- 21 occasions where health promotion advice was given, such as maintaining immunisation status (12), smoking cessation (5) and diet (3).
- 19 instances of advice regarding medication concordance, for example to order replacement inhalers from primary care in a timely manner or change medication regimen. Examples included commencement of gradual withdrawal of corticosteroids (p026) and treatment of oral candidiasis (p026).
- 12 instances where oral care advice was given: rinsing mouth following administration of inhaled medication (7), visiting dentist to pre-empt problems relating to xerostomia (5).
- 9 occasions where patients were advised to send a sputum sample for culture and sensitivity before antibiotics were used.

### Harms

Implementation of the WWADR Profile was neither observed nor reported to be directly harmful. The time taken identifying and responding to problems found may be burdensome to nurses, however, the two nurses involved reported structuring their future care around the Profile. No participant reported negatively on the amount of time spent in clinic or completing the WWADR Profile.

## Discussion

We believe this paper reports the first randomised trial of nurse-led medication monitoring in respiratory medicine. We offer preliminary evidence that formalised and concurrent nurse-led medication monitoring increases the number of patient problems found and responded to; however, interpretation of these findings rests with readers. Using a structured ADR Profile alongside usual care has potential to increase early detection of problems and may therefore ameliorate or pre-empt ADRs and improve patients’ wellbeing and health outcomes [41], [63]–[66]. Identification of problems may prompt the multidisciplinary team to take preventive actions that allow patients to remain well, in accordance with treatment and disease management priorities [67]. It is unlikely that confounding variables account for the differences found, since dynamic randomisation ensured even distribution of variables, reduced biases and maximised the strength of findings [59], [68], [69], and logistic regression identified treatment allocated as the only significant predictor of having more ADR actions taken.

### Medication Monitoring: an Orphan Task

The strength of patient safety initiatives is influenced by the personnel using them and the organisation in which they are used [70]. Whilst nurses, doctors, pharmacists and patients are all eligible to report ADRs, the task of medication monitoring has not been formally embraced by any one profession [24], [60], [71]. Pharmacists have played an integral role in the success of the Finland asthma programme and medication adherence [72], [73], and may be well-placed to monitor medication and review prescriptions [63], [74], [75], but obstacles to their on-going involvement in medication monitoring need to be identified [76]. As inter-professional role boundaries shift and nurses assume roles previously in the medical domain, such as prescribing, there is potential to integrate ADR profiles with nurse-led care. The WWADR Profile helps minimise variation in practice, but organisational uptake should be supported by protected time, learning resources, leadership, dissemination and evaluation [42]. The WWADR Profile may also help overcome some perceived barriers to nurse-led medication monitoring such as inter-professional communication or lack of confidence in knowledge of pharmacology and therapeutics [41], [63]–[65], [77]–[80]. With this support, nurses, as the largest group of front-line healthcare professionals working most closely with service users, are well-placed to play a valuable role in medication monitoring [81], [82]. The Profile itself could be easily transferred across healthcare settings and may alleviate under-reporting of drug-related problems [83], [84].

### Limitations of the Study

As the lasting impact of responding to drug-related harm could not be explored within the resources available to this trial, we cannot ascertain the impact of the initiative on concordance with therapy, enduring amelioration of problems found or long-term harm from administration of the Profile. The Profile prompted health promotion regarding sub-optimal health behaviours, such as poor mouth care or adding salt to meals, and referrals to other professionals. Although some problems were addressed immediately, such as changes in doses of respiratory medicines, clinical gains from nurse-led medication monitoring in outpatient departments depend, in part, on participants following advice to contact other healthcare professionals [60], [61]: for example to treat suspected infections (12/26), obtain overdue vaccinations (12/26) or investigate falls (2/26). Instigation of further treatment or over-treatment would be at the discretion of professionals receiving the referrals, and not directly attributable to the Profile. We were unable to investigate the costs and benefits of referrals. The relatively small sample size precluded detection of small differences, but we feel that our findings indicate sufficient clinical gain to warrant further exploration.

Potential bias in research should always be considered [85]. This study design risked contamination. Over the course of the project, the nurses may have been sensitised to medication monitoring using the WWADR Profile, and unknowingly delivered some aspect of the Profile to the control participants, diluting the observed effect of structured medication monitoring [86]. However, the large effect sizes suggest that contamination was not a major problem.

Blinding during outcome assessment in non-medicinal product [87] or pragmatic trials may be difficult, or impossible, as in this study [88], [89]. Detection bias of outcomes was minimised, but not removed, by a pre-arranged data collection template and the relatively low subjectivity of outcomes [90] such as observation of tremor and statements of advice. We acknowledge that unblinded assessment may increase the risk of bias [91], in line with observers’ expectations [92], suggesting the need for larger multisite investigations. Systematic review indicates that some 3% assessments are likely to be misclassified [93], which would not materially affect interpretation of our study. Observation 1 preceded randomisation.

This trial captures ADR profiles for patients in a single location; accordingly, findings should be generalised cautiously [94], [95]. However, the trial was undertaken in an outpatient nurse-led clinic typical of many district general hospitals, using broad inclusion criteria [95], and patients’ experiences of long-term use of respiratory medicines are not widely reported elsewhere. We did not test alternative instruments or additional appointments. The Hawthorne effect, and social desirability in the nurses’ and participants’ behaviours and communications, cannot be discounted. However, this would have affected both arms equally. Although a good response rate was obtained, a further important consideration is volunteer bias [96]. Volunteer bias could not be directly explored in the current trial, as recruitment rate was high (85%). Findings cannot be generalised to patients too busy or unwilling to participate in research or those viewing their healthcare providers less favourably. Those who took the time to participate in this research may represent a subset of the population who are particularly engaged with their healthcare providers and prescribed regimens.

The literature offers no consensus on identifying and addressing medicine-related harm and adverse events [82]. Many ADRs are subtle and ill-defined, and may resemble pre-existing conditions or ageing. Their aetiology is often multifactorial or uncertain [3]. It was outside the remit of our nurse-led intervention to determine the cause of each problem. Rather, by addressing the diverse problems vulnerable to exacerbation by prescribed medicines [20], [50]–[53], the WWADR Profile works towards incremental optimisation of patients’ health status. In view of the paucity of the evidence base on potential adverse drug reactions [97], [98], to optimise clinical gain, we thought it more important to be thorough and detailed when monitoring for potential medication-related harms, and risk over-ascertainment, than to risk overlooking potentially treatable problems of equivocal aetiology. This approach addresses concerns of public bodies [39], [99]–[101] and participants that “at the moment not enough [is] done to follow-up: how do you know medication’s any good if no one asks you?” (participant 020).

### Interpretation

Patient safety might be improved by concurrent medication monitoring using drug specific checklists [61], [80], [102]–[106]. The WWADR Profile helped nurses structure and prioritise care, and identify previously unsuspected problems. This way, known ADRs may be predicted, pre-empted and addressed, to reduce the burden on long-term medication users. To our knowledge, no previous trials have tested this approach [107]. In this trial, recognising and alleviating symptoms of potential ADRs elicited clinical gains. Completing the WWADR Profile as part of usual care helped protect the time needed to monitor medication. We acknowledge that larger, multisite trials may overturn these findings, but these findings provide the impetus to pursue proactive ADR monitoring research.

### Further Research

This paper suggests that medication monitoring profiles support nurse-led medication monitoring [79], improving the process of care, focusing attention and reducing adverse events in health care [104]–[107]. However, more evidence is needed on the long-term effects of structured medication monitoring on clinical outcomes [41], nursing vigilance, nurses’ workload, nurses’ and service users’ time, and acceptability [20], [39], [40], [108]–[112]. In this small trial, the benefits of medication monitoring for service users outweighed the risks and larger, mulitsite trials of medication monitoring are needed to explore long-term clinical impact. Piloting similar initiatives in different drug groups and healthcare settings, particularly in primary healthcare, where most chronic diseases are currently managed, may enhance patient care.

### Conclusion

Significant differences between intervention and control arms were detected in the recruited sample, supporting our work on antipsychotics [60], [61], [113]. Expanding nursing roles and patient-focused chronic disease management [67] should be encouraged and extended to routine adoption of medicines management policies [114], including standardised medication monitoring profiles [115]. Monitoring should be: accurate, simple, thorough, target-based, capable of detecting insidious and long-term harm, and of improving clinical outcomes [19]. This trial indicates that under-reporting may be improved by structured and standardised ADR profiles [99], [116], [117]. Although many preventable ADRs are due to inadequate patient monitoring [118], many are also due to failure to react to problems found [38]. Actions taken are thus an important focus of this research to ameliorate preventable ADRs [8 38 119–121], and further investigation of ADR Profiles as a strategy to link problem identification with actions is warranted.

## Supporting Information

Table S1 (DOCX)

Checklist S1 **CONSORT checklist Schulz KF, Altman DG, Moher D.** CONSORT 2010 Statement: updated guidelines for reporting parallel group randomised trials. *Trials* 2010;11∶32.
(DOCX)

Protocol S1 **Trial Protocol: Nurse-led medication monitoring.**
(DOC)
