# Supplementary material for: Medication Monitoring in a Nurse-Led Respiratory Outpatient Clinic: Pragmatic Randomised Trial of the West Wales Adverse Drug Reaction Profile
Source: PLoS One. 2014 May 5;9(5):e96682. doi: 10.1371/journal.pone.0096682 (PMC4010491; doi:10.1371/journal.pone.0096682)
Supplement: Table S1 — (DOCX) [file pone.0096682.s001.docx]

**S3 Supplementary table S1. Increase in number of ADR-related actions**

Outcomes of logistic regression analysis of 4 single predictor models

| **Predictor variable** | **B** | **Standard error** | **Wald’s X^2^** | **Significance in model, P value** | **Exponent of B (odds ratio)** | **95% CI of odds ratio** |
| --- | --- | --- | --- | --- | --- | --- |
| **Age (years) (n=54, df=1)** | -0.02 | 0.03 | 0.63 | 0.43 | 0.98 | 0.92 to 1.03 |
| **Sex (male coded as 1 & female coded as 2) (n=54, df=1)** | 0.50 | 0.68 | 0.55 | 0.46 | 1.66 | 0.44 to 6.31 |
| **Length of observation two consultation (minutes) (n=53, df=1)** | 0.02 | 0.06 | 0.14 | 0.70 | 1 02 | 0.91 to 1.16 |
| **Arm allocation (n=54, df=1)** | 2.18 | 0.72 | 9.13 | 0.003 | 8.85 | 2.15 to 36.37 |
| Constant (df=1) | -2.32 | 0.98 | 5.67 | 0.02 | 0.10 |  |

Notes to table:

1. Predictor variables entered one at a time.
2. For the model using arm allocation as the predictor variable:

Omnibus Tests of Model Coefficients *X*^2^ 11.47 (df=1), p = 0.001

Model if term removed, change in -2 log likelihood = 11.47, p=0.001

Nagelkerke R^2^ = 0.27

Cox and Snell R^2^ = 0.19

The Hosmer and Lemeshow test failed because the model had only one variable.
