# Supplementary material for: Medication Monitoring in a Nurse-Led Respiratory Outpatient Clinic: Pragmatic Randomised Trial of the West Wales Adverse Drug Reaction Profile
Source: PLoS One. 2014 May 5;9(5):e96682. doi: 10.1371/journal.pone.0096682 (PMC4010491; doi:10.1371/journal.pone.0096682)
Supplement: Checklist S1 — CONSORT checklist Schulz KF, Altman DG, Moher D. CONSORT 2010 Statement: updated guidelines for reporting parallel group randomised trials. Trials 2010;11∶32. (DOCX) [file pone.0096682.s002.docx]

**S1 Supporting information.** CONSORT checklist Schulz KF, Altman DG, Moher D. CONSORT 2010 Statement: updated guidelines for reporting parallel group randomised trials. *Trials* 2010;**11**:32.

| Section/Topic | Item No | Checklist item |  | Location in paper |
| --- | --- | --- | --- | --- |
| Title and abstract | | | |  |
|  | 1a Identification as a randomised trial in the title | | | Title |
|  | 1b Structured summary of trial design, methods, results, and conclusions | | | Abstract |
| Introduction | | | |  |
| Background and objectives | 2a Scientific background and explanation of rationale | | | Introduction and background |
|  | 2b Specific objectives or hypotheses | | | The study, aim |
| Methods | | | |  |
| Trial design | 3a Description of trial design (such as parallel, factorial) including allocation ratio | | | The study, design |
|  | 3b Important changes to methods after trial commencement (such as eligibility criteria), with reasons | | | Results |
| Participants | 4a Eligibility criteria for participants | | | The study, participants |
|  | 4b Settings and locations where the data were collected | | | The study, setting |
| Interventions | 5 The interventions for each group with sufficient details to allow replication, including how and when they were actually administered | | | The study, intervention |
| Outcomes | 6a Completely defined pre-specified primary and secondary outcome measures, including how and when they were assessed | | | The study, outcome measures & data collection and measurement of outcomes |
|  | 6b Any changes to trial outcomes after the trial commenced, with reasons | | | NA |
| Sample size | 7a How sample size was determined | | | The study, sample size |
|  | 7b When applicable, explanation of any interim analyses and stopping guidelines | | | NA |
| Randomisation: |  |  |  |  |
| Sequence generation | 8a Method used to generate the random allocation sequence | | | The study, randomisation |
|  | 8b Type of randomisation; details of any restriction (such as blocking and block size) | | | The study, randomisation |
| Allocation concealment mechanism | 9 Mechanism used to implement the random allocation sequence (such as sequentially numbered containers), describing any steps taken to conceal the sequence until interventions were assigned | | | The study, randomisation |
| Implementation | 10 Who generated the random allocation sequence, who enrolled participants, and who assigned participants to interventions | | | The study, randomisation |
| Blinding | 11a If done, who was blinded after assignment to interventions (for example, participants, care providers, those assessing outcomes) and how | | | The study, blinding |
|  | 11b If relevant, description of the similarity of interventions | | | NA |
| Statistical methods | 12a Statistical methods used to compare groups for primary and secondary outcomes | | | The study, data analysis |
|  | 12b Methods for additional analyses, such as subgroup analyses and adjusted analyses | | | NA |
| Results | | | |  |
| Participant flow (a diagram is strongly recommended) | 13a For each group, the numbers of participants who were randomly assigned, received intended treatment, and were analysed for the primary outcome | | | Figure 1 |
|  | 13b For each group, losses and exclusions after randomisation, together with reasons | | | Figure 1 |
| Recruitment | 14a Dates defining the periods of recruitment and follow-up | | | The study, participant |
|  | 14b Why the trial ended or was stopped | | | NA |
| Baseline data | 15 A table showing baseline demographic and clinical characteristics for each group | | | Table 1 |
| Numbers analysed | 16 For each group, number of participants (denominator) included in each analysis and whether the analysis was by original assigned groups | | | Figure 1 |
| Outcomes and estimation | 17a For each primary and secondary outcome, results for each group, and the estimated effect size and its precision (such as 95% confidence interval) | | | Results, outcomes |
|  | 17b For binary outcomes, presentation of both absolute and relative effect sizes is recommended | | | Results, outcomes |
| Ancillary analyses | 18 Results of any other analyses performed, including subgroup analyses and adjusted analyses, distinguishing pre-specified from exploratory | | | NA |
| Harms | 19 All important harms or unintended effects in each group (for specific guidance see CONSORT for harms) | | | Harms |
| Discussion | | | |  |
| Limitations | 20 Trial limitations, addressing sources of potential bias, imprecision, and, if relevant, multiplicity of analyses | | | Discussion, limitations of the study |
| Generalisability | 21 Generalisability (external validity, applicability) of the trial findings | | | Discussion, limitations of the study |
| Interpretation | 22 Interpretation consistent with results, balancing benefits and harms, and considering other relevant evidence | | | Discussion, interpretations |
| Other information | | |  |  |
| Registration | 23 Registration number and name of trial registry | | | Abstract |
| Protocol | 24 Where the full trial protocol can be accessed, if available | | | NA |
| Funding | 25 Sources of funding and other support (such as supply of drugs), role of funders | | | Funding |
