# Supplementary material for: Medication Monitoring in a Nurse-Led Respiratory Outpatient Clinic: Pragmatic Randomised Trial of the West Wales Adverse Drug Reaction Profile
Source: PLoS One. 2014 May 5;9(5):e96682. doi: 10.1371/journal.pone.0096682 (PMC4010491; doi:10.1371/journal.pone.0096682)
Supplement: Protocol S1 — Trial Protocol: Nurse-led medication monitoring. (DOC) [file pone.0096682.s003.doc]

**S2 Trial Protocol: Nurse-led medication monitoring**

This protocol was developed to follow the Standard Operating Procedures of the West Wales Organisation for Rigorous Trials in Health.

Full title of trial

**Nurse-led medication monitoring and adverse events in a respiratory medicine outpatient department: a single site parallel group pragmatic randomised controlled trial (RCT) of the West Wales Adverse Drug Reaction (WWADR) Profile for Respiratory Medicine.**

Allocation by concealment, stratified randomisation by West Wales Organisation for Rigorous Trials in Health (WWORTH).

IRCTN trial registration number: ISRCTN10386209

Trial summary

Background: Adverse drug reactions (ADRs) associated with respiratory medicine can cause preventable patient morbidity and mortality. Medication-monitoring may curtail unnecessary patient harm but is, however, limited in practice.

Objectives: To assess the clinical impact nurse-led medication monitoring.

Design: A single site parallel group pragmatic randomised controlled trial (RCT) of the West Wales ADR (WWADR) Profile for Respiratory Medicine with a comparator and intervention group assigned by stratified randomisation by WWORTH.

Setting: Nurse-led respiratory clinics in an outpatient department of a large teaching hospital.

Participants: Patients attending the respiratory clinics with a chronic respiratory condition for which they receive one of more of: corticosteroids, bronchodilators, theophylline and / or leukotriene receptor antagonists.

Intervention: The WWADR Profile for Respiratory Medicine.

Primary outcome measure: Differences in number of problems actioned.

Underlying true outcome measure: Differences in number of problems detected.

Contact applicants

Marie Gabe

College of Human and Health Sciences,

Swansea University,

Singleton Park. SA2 8PP.

Sue Jordan, Reader,

College of Human and Health Sciences,

Swansea University,

Singleton Park.SA2 8PP.

01792 518541

Problem to be addressed

To limit the patient health burden of the known ADRs in respiratory medicine. To flag ownership of medication monitoring in healthcare by nurses.

Principal research question

Does concurrent and formal monitoring of respiratory medication using the WWADR Profile for Respiratory Medicine in addition to ‘usual’ care increase the number of detected and actioned problems compared to patients who receive ‘usual’ nursing care alone?

Need for trial

As a culture of patient safety and leadership gain increasing priority (DoH, 2000; WAG 2005; NHS, 2008; Sammer et al., 2010) paucity of medication monitoring leaves patients vulnerable to the ill-effects of medication (Pirmohamed et al., 2004; Thomsen et al., 2007; Onder et al., 2008; Bravar et al., 2009). To date there is little work exploring the clinical impact of ADR checklists and the roles of healthcare professionals in medication monitoring (Mjörndal et al. 2002).

Literature searches using electronic databases: CINAHL, PubMed, SwetsWise, Zetoc, and the Cochrane Library were undertaken. Databases were searched from October 2009-July 2010 using the following search terms, truncated and in combinations: AEs, ADRs, medication monitoring, nurse-led medication monitoring, pharmacovigilance, and professional roles. A supplementary manual search was also conducted with eminent authors being reviewed. Only English language publications based on ‘all adult’ age group data, without time limit, were reviewed (Gabe et al., [in press]).

Previously, nurse-led medication monitoring in psychiatric medicine highlighted a need for proactive medication monitoring (Jordan et al., 2002; Jordan et al., 2009; Gabe et al., [in press]). Raising awareness of ADRs by healthcare professionals, whilst minimising reporting confusion and under-reporting (Winterstein et al. 2002, Kanjanarat et al. 2003. Brvar et al. 2009; Davies et al., 2010), should help unite ADR detection with action. Elsewhere, the patient perspective has been highlighted as a neglected source of ADR data (Davies et al., 2010). One questionnaire has been located in respiratory medicine, exploring the ADRs of a single drug (Foster et al. 2006a, 2006b) in what is often a poly-drug treatment regimen (The Global Initiative for chronic Obstructive Lung Disease GOLD, 2008). Further intervention research is thus essential (Nguyen et al. 2001, Gurwitz et al. 2003, Forster et al. 2005; Cereza et al., 2010) to explore the clinical effectiveness of a single ADR profile that explores the various ADRs of a poly-drug respiratory regimen.

Timeliness of the trial

In an ageing population the prevalence of asthma and COPD is high. An emphasis on long-term disease management in the community (Worth et al., 2010) highlights the increased reliance upon pharmaceutical therapy to treat and manage disease. Treatment should occur with minimal side-effects (Rabe et al., 2007) and unless known ADRs are routinely and actively monitored under-reporting and the patient burden of treatment will continue to go unnoticed. Research is now needed to evaluate the clinical impact of proactive nurse-led medication monitoring.

Risks and benefits

We do not envisage any physical or psychological risk to individuals involved in this research. However, the trained staff in the outpatient unit are prepared to provide counselling and support as needed to the participants.

This project aims to provide long-term benefits to patients. By continuing to provide the usual and standard care of the department, the participants’ health will continue to be safeguarded. All identified anxieties and health burdens will be referred as necessary and consultants will see patients with any new medical problems highlighted.

How will findings be used?

Results of the trial will be published in a thesis and submitted for publication in journals. The doctors, nurses and patients involved in this study will be provided with an executive summary of the study. If successful, the findings will be used to further explore the clinical impact of nurse-led medication monitoring in respiratory medicine in larger intervention studies, multi-site cluster or stepped wedge trial of medication monitoring.

Trial design

The WWADR profile for respiratory medicine has been developed and tested for inter-rater reliability. It is now being evaluated for clinical effectiveness in a single site parallel-group pragmatic RCT using stratified random allocation to the comparator and intervention groups. Participants are observed on two occasions by a researcher during their usual nurse-patient consultations. Participants are told that in addition to standard and usual care of the hosting clinic they may be asked some extra questions and measurements, such as blood pressure and girth, may be taken for research purposes.

At the end of each clinic in the first round of observations, participant study numbers will be passed to WWORTH (the local registered clinical trials unit) over the telephone for randomisation into intervention or comparator group for the second round of observations. The outcome of randomisation will then be used to assign participants to a clinic day, Wednesday or Friday. The researcher will indicate which day the participant is to attend on the patients’ letter used to book appointments by the outpatients’ clerk. The exact date of appointments will not be provided at that time but will be provided to participants one month before they are due in clinic. The interval between the first and second round of observations will be typically some 3-6 months and is dictated by the nurses’ clinical judgment, based on participants’ needs (table 1). Background changes during this lag time could affect the accuracy of randomisation stratification at observation one, for example participants could have their number of respiratory medicines increased or decreased. However, the interval between observations is necessary and representative of the usual nursing offered in the clinic.

Participants are informed of their appointment by post one month before they are due in clinic, the researcher, using patient hospital number, will track their return to clinic using the electronic patient information system in the hosting clinic. The WWADR profile will be implemented at the second round of observations, intervention group only.

Table 1. Participant observations

|  | Clinic visit 1 data collection | Interval between clinic visits (typically 3-6 months) | Clinic visit 2 data collection |
| --- | --- | --- | --- |
| Intervention  nurse-led clinic 1 | Usual care  Observation* of 25-50 consultations | Usual care | Usual care + intervention monitoring  Observation* of 25-50 consultations |
| Comparator  nurse-led clinic 2 | Usual care  Observation* of 25-50 consultations | Usual care | Usual care  Observation* of 25-50 consultations |

Trial interventions

Both groups receive the usual and standard care of the hosting clinic. The intervention group, in addition, upon second observation will be asked to respond to the WWADR Profile for Respiratory Medicine. It is a five sectioned profile that records medication used by patient, vital signs, and asks a series of questions about their medication and several observations along with checking when tests were last done.

Group allocation

Stratified random sampling based on age (median 61 years), day of initial clinic and number of respiratory drugs used (median two) by the WWORTH. Median data were calculated during the pilot testing of WWADR2 (n=25). The intervention participants will be assigned to a Friday clinic in the future and the comparator group to a Wednesday clinic in the future. The questions asked during the randomisation call will be;

- Study ID number (001-060)
- Age
- Day of initial clinic
- Number of respiratory drugs taken
- Confirmation that the inclusion and exclusion criterion apply
- Participant initials (used to validate the trial number).

Bias

Being mindful of the constraints of the hosting clinics, the study was not able to randomly allocate nurses, as users of the WWADR profile. However, to limit the degree of sensitisation of the nurses, the two clinics, Wednesday afternoon and Friday morning, were assigned as a control and intervention clinic respectively before phase three begun. This division of clinics was based on discussion with the clinical and academic team involved in the trial. The aim of this was to not only limit the incorrect use of profile between the groups but to provide the maximal time interval between the use of the profile in the intervention group and then in the comparator group.

Inclusion and exclusion criteria

Inclusion Criteria:

- Clients of the outpatient respiratory clinic.
- Existing chronic respiratory condition.
- Participants will need to be currently prescribed and receiving at least one of:
  - Bronchodilators
  - Theophylline
  - Corticosteroids
  - Leukotriene antagonists

Exclusion Criteria:

- Patients aged 16 or under.
- Lack of fluency in English or Welsh.
- Patients will be screened by trained physicians and/or nurses to ensure they are well enough to participate in this project, and will be excluded as necessary by their clinicians:
  - - Patients considered lacking the capacity to consent.
    - Patients experiencing undue stress or vulnerability.

Duration of intervention

Typically patients are assigned a 15-20 minute slot in clinic and will be observed during two separate interviews, typically some three-six months apart. The intervention was piloted elsewhere in the study during measures of inter-rater reliability (phase two). The intervention took between10-45 minutes to complete (average 25) excluding time spent checking tests.

Follow-up

All patients will continue with their usual care follow up, 3-6 monthly. Any problems detected will be noted, discussed with clinic nurses and passed to consultant physicians, if necessary. No research follow-up is planned. As the researcher is the observer and the participants are being observed in their usual nurse-patient appointments with their nurse, the nurse as the primary carer is responsible for identifying adverse events. However, should the researcher need to, any problems highlighted will be relayed in a timely manner to the nurses who then may, in turn, wish to contact the patients’ consultant.

Outcome measures

The primary outcome, used to predict sample size, is the number of problems actioned by nurses. The underlying true outcome is the number of problems detected.

Measurement of outcomes measures

The WWADR and field notes will be used to record the number of problems and identify action taken in all participants at both observations. The ‘severity’ of the problems detected is subjective. For example, one may consider postural hypotension to be more ‘severe’ than failure to take medication as prescribed or mouth care. The researcher, whilst providing descriptive accounts of the problems identified and action taken, does not assume any priority for data purposes. A problem was therefore seen as a problem regardless of its severity.

Sample size

In previous work, 14/20 service users receiving usual care and 1/20 service users responding to a structured monitoring instrument had no adverse drug reactions actioned by their nurses (Jordan 2002, Jordan et al 2002). A sample of 34, 17 in each group, is sufficient to detect this difference in incidence of nursing actions. However, this calculation took no account of any possible clustering effects that could arise due to team working, and we would expect to increase the sample size, initially to 40 (Uitenbroek, 1997). The power is 90% and significance 0.01. To allow for potential losses to follow up the sample is inflated to 30 in each group, 60 participants in total.

At participant 25, using the means and SDs, we will compare data between phases two and three. Phase two involved inter-rater reliability testing (May-September 2010) using the WWADR profile. Accordingly, recruitment will be increased if necessary but capped within the limitations of a PhD study.

Duration of recruitment

The study was initially introduced to prospective participants both verbally and with information sheets up to some seven months previously (May 2010) during an earlier phase of the research. The system of randomisation will be in place (January 2011) for active recruitment to begin.

Recruitment rate

Patients attending either the Wednesday or Friday clinics will be initially approached and introduced to the trial by their nurse or the researcher. Typically 8-15 patients attend the weekly clinics. The numbers of ineligible patients vary due to pulmonary rehabilitation assessments (every 12weeks) and patients with tuberculosis (TB) attending clinic. Based on phase two data, 18 ineligible patients attended clinic over a four month period (June 2010-September 2010). 16 patients had TB and 2 patients were too unwell. From phase two recruitment (allowing for DNA, ineligible patients) and patient feedback we suggest that we may recruit up to 5 participants a week.

Compliance

Patients attending clinic will be able to withdraw, but not to decline to comply with the intervention. They will not be asked to comply on leaving the clinic. If clinic nurses become too busy, they may decline to comply, and we would hope to observe the participant in a future clinic. The profile may need to be delivered over 2 appointments.

Loss to follow-up

To allow for possible loss to follow-up we plan to recruit at least 30 into each arm. Previously 20 in each arm were sufficient.

Trial sites

The trial will be conducted in an outpatient department of Singleton Hospital, a large teaching hospital in Wales that serves a catchment area extending to a population of around 600,000. The hospital bed capacity is almost 600 and hosts a number of adult and paediatric outpatient services including physiotherapy, cardiac and pulmonary rehabilitation along with specialised clinics such as dermatology, ophthalmology, haematology and endocrinology, including respiratory clinics. Across the whole NHS Health Board approximately 17,000 staff are employed. The hosting clinic is a nurse-led respiratory clinic which runs twice each week.

Analyses

Problems and actions will be recorded descriptively. Cross-tabulations and comparison of key variables will be undertaken to explore the data. Data will be statistically analysed and reported in the most powerful way using SPSS version 16.

Economics

Benefits may include improving patient health through structured medication monitoring. The projected costs are the extra time spent completing the profile and actioning problems along with costs associated with any necessary training. However, as the profile has guiding instructions it is felt that training needs will be minimal.

Quality assurance: To follow WWORTH standard operating procedures

Budget: Studentship. Funded by RCBC Wales

Trial team

The trial team are from two main arenas, there is academic and clinical support and expertise, including at the trial site. They include:

Dr. Sue Jordan, Dr. Fiona Murphy, Prof. Ian Russell, Prof. Gary Rolfe, Dr. Gwyneth Davies, Ms Michelle Davies and Ms Linzi Johnstone.

WWORTH, Dr. Kym Thorne, Prof. Ian Russell.

Dr. Daphne Russell will undertake randomisation.

Flow-chart Patient approach, recruitment and consent flow chart.

| Written information |  | Leaflet given out count:  From reception n=  From researcher n=  From clinic nurses n= |  |  |
| --- | --- | --- | --- | --- |
|  |  |  |  | Consult or nurse excluded patients n= |
| Approach |  | Expression of interest forms n=  Direct approach from patient n=  Approach by researcher n=  Approach by clinic nurses n= |  |  |
|  |  |  |  | Decline n=  Ineligible n= |
| Consent by researcher |  | Consented patients n= |  |  |
|  |  |  |  | Withdrawn from study n= (reasons)  Records removed from study n= |
| Proceed to study Phase Three: Same day contact One with participants |  | Completed round one and randomised n= |  |  |
|  |  |  |  | Withdrawn from study n= (reasons)  Records removed from study n=  Failure to attend n=  Become ineligible (reasons e.g. too ill) n= |
| Proceed to Phase Three: Contact Two with participants |  | Retained patients completing round two n= |  |  |
|  |  |  |  | Withdrawn from study n=  Records removed from study n= |
| Completion of Phase Three |  | Total completed at both contacts n=  Total number of dropouts n= |  |  |
|  |  |  |  |  |
|  |  | Final data n= |  |  |

References:

Brvar M., Fokter N., Bunc M. & Mozina M. (2009) The frequency of adverse drug reaction related admissions according to method of detection, admission urgency and medical department speciality. BMC Clinical Pharmacology **9** (8)

Cereza, G., Agustί, A., Pedrós, C., Vallano, A., Aguilera, C., Danés, I., Vidal, X., and Arnau, J.M. (2010) Effect of an intervention on the features of adverse drug reactions spontaneously reported in a hospital. European Journal of Clinical Pharmacology. 66, 937-945

Davies, E.C., Green, C.F., Mottran, D.R., and Pirmohamed, M. (2010) Interpreting adverse drug reaction (ADR) reports as hospital patient safety incidents. Brithish Journal of Clinical Pharmacology. **70** (1) 102-108

Department of Health (2000) An Organisation with a Memory: Report of an Expert Group on Learning from Adverse Events in the NHS. The Stationery Office, London

Forster AJ, Murff HJ, Peterson JF, Gandhi TK, Bates D (2005) Adverse drug events occurring following hospital discharge. Journal of General Internal Medicine. **20** (4) 317-323.

Foster J.M., Aucott L., Van Der Werf R.H.W., Meijden M.J.V., Schraa G., Postma D.S. & Molen T.V. (2006a) Higher patient perceived side effects to higher daily disease of inhaled corticosteroids in the community: A cross-sectional analysis. Respiratory Medicine **100** (8), 1318-1336.

Foster J.M., Sonderen E.V., Lee A.J., Sanderman R., Dijkstra A., Postma D.S. & Molen T.V. (2006b) A Self rating scale for patient-perceived side effects of inhaled corticosteroids. Respiratory Research **7,**131

Gabe, M.E., Davies, G.A., Murphy, F.M., Davies, M., Johnstone, L., Jordan, S.E. (in press) Adverse drug reactions: Treatment burdens and nurse-led medication monitoring. Journal of Nursing Management. Accepted 12/10/2010

Gurwtiz, J.H., Field, T.S., Harrold, L.R., Rothschild, J., Debellis, K., Seger, A.C., Cadoret, C., Fish, L.S., Garber, L., Kelleher, M., and Bates, D.W. (2003) Incidence and Preventability of Adverse Drug Events Among Older Persons in the Ambulatory Setting. JAMA. **289** (9) 1107-1116

Jordan S. (2002) Managing Adverse Drug Reactions: An Orphan Task. Developing Nurse-Administered Evaluation Checklists Journal of Advanced Nursing **38** (5) 437-48

# Jordan S., Tunnicliffe C., Sykes A. (2002) Minimising Side Effects: The clinical impact of nurse-administered ‘side effect’ checklists. Journal of Advanced Nursing. 37 (2) 155-65

# Jordan S., Jones R., Sargeant M. (2009) Adverse drug reactions: managing the risk. Journal of Nursing Management. 17 175-84

Kanjanarat P., Winterstein A.G., Johns T.E., Hatton R.C., Gonzalez-Roothi R. & Segal R. (2003) Nature of preventable adverse drug events in hospitals: a literature review. American Journal of Health-System Pharmacy **60** (17), 1750-1759.

Mjörndal T., Boman M.D., Hägg S., Bäckström M., Wiholm B., Wahlin A. & Dahlqvist, R. (2002) Adverse drug reactions as a cause for admissions to a department of internal medicine. Pharmacoepidemiology and Drug Safety **11** (1), 65-72.

National Health Service (2008) 1000 Lives Campaign. Available at: <http://www.wales.nhs.uk/sites3/home.cfm?orgid=781> [accessed: 01/12/2009]

Nguyen T.V., Hillman K.M. & Buist M.D. (2001) Adverse events in British hospitals. Prevention strategies, not epidemiological studies, are needed. British Medical Journal **322,** 1425.

Onder G., Pedone C., Landi F., Cesari M., Vedova C.D., Bernabei R. & Gambassi G. (2008) Adverse Drug Reactions as Causes of Hospital Admissions: Results from the Italian Group of Pharmacoepidemiology in the Elderly (GIFA). Journal of the American Geriatrics Society **50** (12), 1962-1968.

Pirmohamed M., James S., Meakin S., Green C., Scott A.K., Wallet T.J., Farrar K., Park B.K. & Breckenridge A.M. (2004) Adverse drug reactions as cause of admission to hospital: prospective analysis of 18820 patients. British Medical Journal **329** (7456), 15-19.

Rabe, K.F., Hurd, S., Anzueto, A., Barnes, P.J., Buist, S.A., Calverley, P., Fukuchi, Y., Jenkins, C., Rodriguez-Riosin, R., Van Weel, C., and Zielinski, J. (2007) Global Strategy for the Diagnosis, Management, and Prevention of Chronic Obstructive Pulmonary Disease. American Journal of Respiratory and Critical Care Medicine. **176** (6) 532-555.

Sammer, C.E., Lykens, K., Singh, K.P., Mains, D.A., and Lackan, N.A. (2010) What is Patient Safety Culture? A Review of the Literature. Journal of Nursing Scholarship. **42** (2) 156-165

The Global Initiative for Chronic Obstructive Lung Disease (GOLD) (2008) Global Strategy for the Diagnosis, Management and Prevention of COPD. Available at: <http://www.goldcopd.com/Guidelineitem.asp?l1=2&l2=1&intId=2003>

Thomsen L.A., Winterstein A.G., Haugbølle L.S. **& Melander A.** (2007) Systematic Review of the Incidence and Characteristics of Preventable Adverse Drug Events in Ambulatory Care. The Annals of Pharmacotherapy **41** (9), 1411-1426.

Uitenbroek, D. G. (1997) "SISA-Binomial", Available: <http://home.clara.net/sisa/binomial.htm> . (Accessed: 2002, January 1). (SISA (Simple Interactive Statistical Analysis) <http://home.clara.net/sisa/sampshlp.htm> accessed July 13th 2009

Welsh Assembly Government (WAG) 2005 Designed for Life: creating world class Health and Social Care for Wales in the 21^st^ Century. Welsh Assembly Government, Cardiff

Winterstein A.G., Sauer B.C., Hepler C.D. & Poole C. (2002) Preventable drug-related hospital admissions. Annals of Pharmacotherapy **36,** 1238-1248.

Worth, A., Pinnock, H., Flethcer, M., Hoskins, G., Levy, M.L., and Sheikh, A. (2011) Systems for the management of respiratory disease in primary care – an international series: United Kingdom. Primary Care Respiratory Journal. **20** (1), 23-32.
